# Molecular Evolution and Functional Divergence of Stress-Responsive Cu/Zn Superoxide Dismutases in Plants

**DOI:** 10.3390/ijms23137082

**Published:** 2022-06-25

**Authors:** Guozhi Zhou, Chaochao Liu, Yuan Cheng, Meiying Ruan, Qingjing Ye, Rongqing Wang, Zhuping Yao, Hongjian Wan

**Affiliations:** 1 State Key Laboratory for Managing Biotic and Chemical Threats to the Quality and Safety of Agro-Products, Zhejiang Academy of Agricultural Sciences, Hangzhou 310021, China; zhougz@zaas.ac.cn; 2 Institute of Vegetables, Zhejiang Academy of Agricultural Sciences, Hangzhou 310021, China; chengyuan@zaas.ac.cn (Y.C.); ruanmy@zaas.ac.cn (M.R.); yeqj@zaas.ac.cn (Q.Y.); wangrq@zaas.ac.cn (R.W.); yaozp@zaas.ac.cn (Z.Y.); 3 School of Biotechnology, Jiangsu University of Science and Technology, Zhenjiang 212021, China; qdliuchaohi@163.com; 4 China-Australia Research Centre for Crop Improvement, Zhejiang Academy of Agricultural Sciences, Hangzhou 310021, China

**Keywords:** reactive oxygen species, abiotic, expression profiles, subcellular localization, phylogenetic relationship

## Abstract

Superoxide dismutases (SODs), a family of antioxidant enzymes, are the first line of defense against oxidative damage and are ubiquitous in every cell of all plant types. The Cu/Zn SOD, one of three types of SODs present in plant species, is involved in many of the biological functions of plants in response to abiotic and biotic stresses. Here, we carried out a comprehensive analysis of the Cu/Zn SOD gene family in different plant species, ranging from lower plants to higher plants, and further investigated their organization, sequence features, and expression patterns in response to biotic and abiotic stresses. Our results show that plant Cu/Zn SODs can be divided into two subfamilies (group I and group II). Group II appeared to be conserved only as single- or low-copy genes in all lineages, whereas group I genes underwent at least two duplication events, resulting in multiple gene copies and forming three different subgroups (group Ia, group Ib, and group Ic). We also found that, among these genes, two important events—the loss of introns and the loss of and variation in signal peptides—occurred over the long course of their evolution, indicating that they were involved in shifts in subcellular localization from the chloroplast to cytosol or peroxisome and underwent functional divergence. In addition, expression patterns of Cu/Zn SOD genes from *Arabidopsis thaliana* and *Solanum lycopersicum* were tested in different tissues/organs and developmental stages and under different abiotic stresses. The results indicate that the Cu/Zn SOD gene family possesses potential functional divergence and may play vital roles in ROS scavenging in response to various stresses in plants. This study will help establish a foundation for further understanding these genes’ function during stress responses.

## 1. Introduction

Reactive oxygen species (ROS) play an important role in plant growth, development, and, especially, responses to biotic and abiotic environmental stimuli. The major members of the ROS family are free radicals (O^2−^, OH^•^) and non-radicals (H_2_O_2_ and ^1^O_2_). These free radicals can cause extensive damage to protein, DNA, and lipids and, thereby, affect normal cellular functioning [1,2]. However, in the long process of evolution, plants have developed multiple defense mechanisms to alleviate the damage caused by these free radicals and maintain redox homeostasis. Among them, two kinds of antioxidant machinery have been identified; one involves enzymatic components comprising superoxide dismutase (SOD), ascorbate peroxidase (APX), guaiacol peroxidase (GPX), glutathione-S-transferase (GST), and catalase (CAT); and the other involves non-enzymatic low-molecular-weight compounds, such as ascorbic acid (AA), reduced glutathione (GSH), α-tocopherol, carotenoids, phenolics, flavonoids, and proline [3,4,5].

Superoxide dismutase (SOD) is a vital component of the oxidative stress tolerance system in plants, microorganisms, and animals [6,7]. The SODs, a family of the metalloenzymes omnipresent in all aerobic organisms, act towards the degradation of O^2−^ free radicals to O_2_ and H_2_O_2_. Under environmental stresses, SOD forms the first line of defense against ROS-induced damage. SOD can catalyze the removal of O^−2^ by dismutating it into O_2_ and H_2_O_2_. This removes the possibility of OH formation through the Haber–Weiss reaction [8]. At present, based on the metal ion they bind and subcellular distribution, SODs are mainly categorized into iron SODs (Fe-SODs, localized in chloroplasts), manganese SODs (Mn-SODs, localized in mitochondria,), and copper/zinc SODs (Cu/Zn SODs, localized in cytosol, peroxisomes, and chloroplasts) [9]. Fe-SOD has been localized in chloroplasts, while Mn-SOD is found in mitochondria. The Cu/Zn SODs are produced in three different cell organelles, including chloroplast, peroxisomes, and cytoplasm [9]. Cu/Zn SODs are widely present in prokaryotic and eukaryotic organisms and were first cloned from *Photobacterium leiognathi* [10,11]. Cu/Zn SOD, present in eukaryotic cells, has been found to be sensitive to cyanide and is located in the form of a dimer; however, Fe-SOD and Mn-SOD are not sensitive to cyanide [12].

It has been reported that, under various environmental stresses, SOD activity can increase in various plant species, including drought and metal toxicity [13]. For example, SOD activity increases were detected in three different cultivars of *Phaseolus vulgaris* and *Oryza sativa* in response to drought stress [14,15,16]. Increased SOD activity was found under drought stress in the leaves of white clover; viz., *Trifolium repens* L. [17]. SOD activity was also found to be heightened under salt stress in many plants, such as chickpea [18] and tomato [19]. Presently, the increases in SOD concentration have been used to improve oxidative stress tolerance in various plant species [20]. Therefore, it has also been reported that SOD production by different plant species can be used as a tool for screening the stress-toleration capacities of plant and microbe species [21].

In addition, some researchers have also reported that Cu/Zn SOD genes are expressed in plant growth and development and in response to different abiotic stresses, including heat, cold, drought, and salinity. For instance, two Cu/Zn SOD genes isolated from tomato leaves were found to be distributed on different chromosomes [22,23]. Subsequently, the expression of these two Cu/Zn SOD genes in tomato organs during leaf growth and fruit ripening were reported [24]. Wu et al. reported that multiple abiotic stresses were conferred in transgenic Arabidopsis by the overexpression of a Cu/Zn SOD from *Puccinellia tenuiflora* (Put Cu/Zn SOD) [25]. Recently, researchers have found that enhancements in antioxidative defense capacity are usually used to improve stress tolerance in plants. For example, overexpression of the Cu/Zn SOD gene from *Arachis hypogaea*, *Kandelia candel,* and wheat could increase the resistance to salt stress, resulting in a higher antioxidative defense capacity [26,27,28]. The overexpression of *SaCu/ZnSOD* in *Sedum alfredii* could confer tolerance to oxidative stress in *Arabidopsis* by increasing antioxidative defense capacities [29]. Further, Perl et al. [30] found that the overexpression of Cu/Zn SODs in potato resulted in increased tolerance to oxidative stress in transgenic plants. In addition, several recent studies have indicated that SOD plays an important role in hormone and insecticide stresses. For example, Bernal et al. [31] detected that the expression level of cytosolic Cu/Zn SODs had increased after 1 day of auxin treatment in tomato.

In recent years, with the availability of complete genome sequences from different plant species, a number of members of the SOD gene family have been systematically identified in some plant species at the genome level, such as *A. thaliana* [32], tomato [33], *Cucumis sativus* [34], *Gossypium hirsutum*, *G. arboreum*, and *G. raimondii* [35,36,37]. However, little has been reported about the characterization, expression patterns, and phylogeny of the Cu/Zn SOD gene family. From lower to higher plant species, abiotic stresses affect plant growth and development. Plants’ abilities to resist abiotic stresses are evolving. In the present study, in order to explore evolutionary relationships of Cu/Zn SODs in plant species, we characterized and identified Cu/Zn SOD genes in different plant species ranging from lower to higher plants. The results showed a high conservation of Cu/Zn SOD genes in different plant species. Two groups of Cu/Zn SODs were found with different evolutionary modes; i.e., group I underwent two duplication events, while group II appeared to be conserved only as a single gene. Furthermore, loss of introns and loss of and variation in signal peptides occurred in group I, which demonstrated structural feature variation, indicating that shifts in subcellular localization from the chloroplast to the cytosol or peroxisome occurred for group I during evolution. Additionally, expression levels of Cu/Zn SODs from *A. thaliana* and *S. lycopersicum* in response to cold, salt, drought, and heat were increased, indicating that these genes play vital roles under abiotic stress conditions.

## 2. Results

### 2.1. Plant Cu/Zn SODs: A Relatively Small Gene Family with Several Members

**Figure 1:**
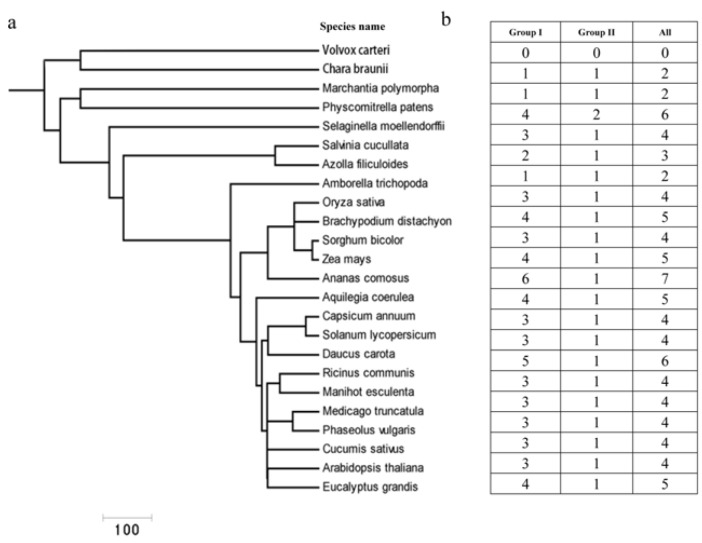
Retrieval and identification of Cu/Zn SOD genes in different plant species ranging from green algae to higher plants. (**a**) Phylogeny of different plant species from green algae to higher plant species. (**b**) The distribution of Cu/Zn SOD genes in representative species.

We used the BLASTP method and a hidden Markov model (HMM) algorithm to search for the Cu/Zn SOD genes in a comprehensive dataset containing different plant species ranging from lower to higher plants [38]. In total, 87 sequences were retrieved from 21 different plant species (Supplementary Table S1). Among them, Cu/Zn SOD genes were found to be present in major lineages of green plants, including Charophyte (e.g., *C. braunii*), Bryophyte (*Marchantia polymorpha*, *Physcomitrella patens*), Pteridophyta (*Selaginella moellendorffii*), and angiosperms (e.g., *A. thaliana*, *Oryza sativa*) (Figure 1a,b). The copy number of Cu/Zn SOD genes varied considerably between the different plants, ranging from two in the green alga *C. braunii* to four in *A. thaliana* (eudicot), with the highest number of seven in *Aquilegia coerulea* (eudicot). No Cu/Zn SOD proteins were detected in the draft genomes of the Chlorophyte *Volvox carteri*. Further investigation revealed that the copy number variation in different plants was mainly due to the difference in the phylogenetically defined group I (Figure 1b); in the other group (II), only one or two copies were found (e.g., the copy numbers were nearly constant).

### 2.2. Division of Cu/Zn SOD Proteins into Two Groups Based on Domain Architecture and Conserved Residues

**Figure 2:**
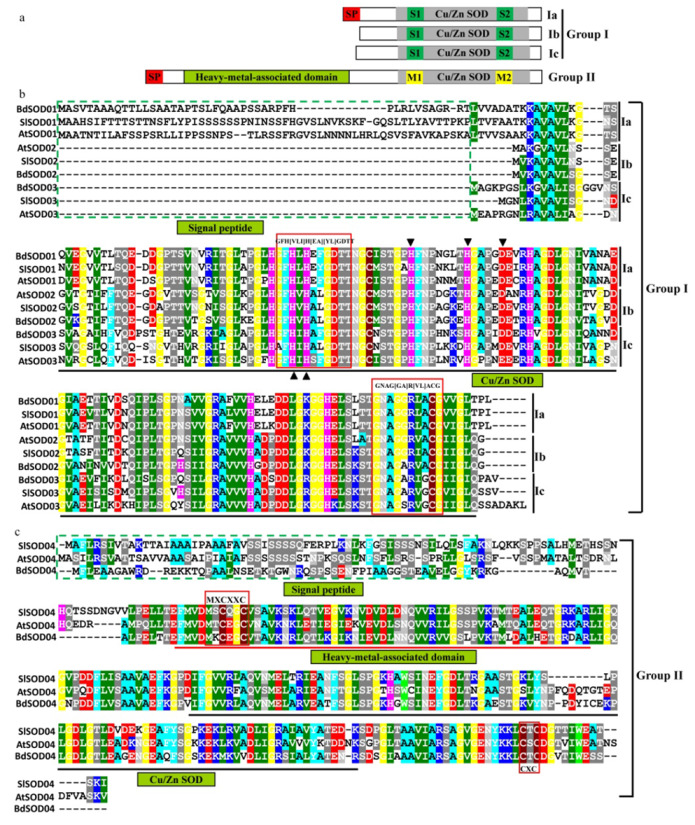
The domain architecture and sequence conservation of plant Cu/Zn SOD proteins. (**a**) Schematic representation of domain architecture of each type of Cu/Zn SOD protein. (**b**) Multiple sequence alignment of the deduced amino acid sequences of Cu/Zn SOD proteins. The conservation domain of Cu/Zn SOD without a heavy metal-associated domain is underlined. Two conserved Cu/Zn SOD signatures (GFH[VLI]H[EA][YL]GDTT and GNAG[GA]R[VL]ACG) are indicated by red boxes. The metal-binding sites for Cu^2+^ and Zn^2+^ are marked with regular and inverted triangles, respectively. The signal peptide is marked by a green box. (**c**) Multiple sequence alignment of the deduced amino acid sequences of Cu/Zn SOD with the heavy metal-associated domain. The conserved domains (“heavy metal-associated domain” and “Cu/Zn SOD”) were underlined (red and black, respectively). The conserved metal-binding motifs (MXCXXC and CXC) are marked by red boxes. The signal peptide is marked by a green box.

All the Cu/Zn SOD proteins were divided into two groups (group I and group II) based on whether they contained the “heavy metal-associated domain” or not. Group I was further categorized into subgroups based on the possession of other conserved domains or motifs. For group I, one subgroup (Ia) had an N-terminal signal peptide, which was indicative of secretion from or localization to organelles; another subgroup (Ib) lacked this domain and appeared to be localized to the cytosol; the third group (Ic) possessed a relatively long N-terminal sequence. All three of these subgroups possessed two conserved Cu/Zn SOD signatures (S1 and S2). The members of group II contained, as well an N-terminal signal peptide, a heavy metal-associated domain and two conserved metal-binding motifs (designated M1 and M2) (Figure 2a).

The S1 signature is characterized by a G-F-H-[VLI]-H-[EA]-[YL]-G-D-T-T motif and the S2 signature by a G-N-A-G-[GA]-R-[VL]-A-C g motif. The arrangement of these two signatures was conserved in group I Cu/Zn SOD proteins (Figure 2b). In addition, several amino acids that were conserved across all Cu/Zn SOD proteins and multiple diagnostic residues restricted to each subgroup were also observed. Among them, the two and three metal-binding sites for Cu^2+^ and Zn^2+^ were conserved in the tested plant species and are marked with regular and inverted triangles, respectively. These conserved residues are potentially important for the structural conformation of these proteins, whereas differences may affect substrate preferences. For group II, an N-terminal signal peptide, indicative of localization to chloroplasts, and a heavy metal-associated domain were conserved. Two conserved metal-binding motifs (M-X-C-X-X-C and C-X-C) were found to be conserved, where X is a variable amino acid residue.

### 2.3. Phylogenetic Analyses Support the Two Groups of Cu/Zn SOD Proteins and Reveal Lineage-Specific Expansions

**Figure 3:**
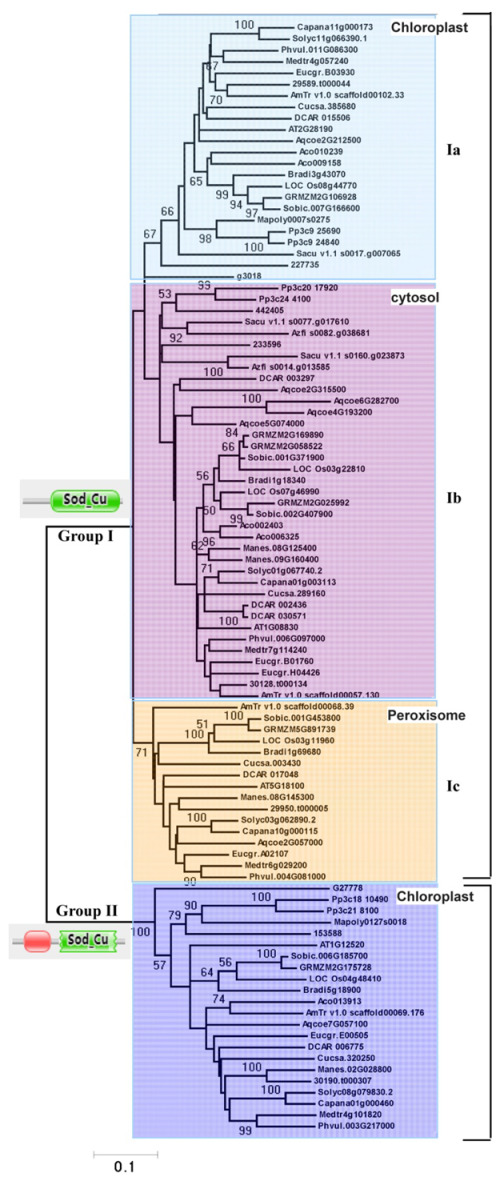
Phylogenetic analysis of the Cu/Zn SOD proteins. Representative phylogenetic tree based on the NJ method. Statistical support is indicated at the nodes. Only statistical support values >50% are shown. Accession numbers of the proteins used in this tree can be found in Suplementary File S1.

To explore the phylogenetic relationships of plant Cu/Zn SOD genes, a phylogenetic tree with full-length sequences from different species was produced using the NJ method. Based on our phylogenetic analyses and subcellular localization prediction results (Figure 3), all the plant Cu/Zn SOD genes can be divided into two major clades, designated group I and group II. Therefore, the two groups of Cu/Zn SOD genes identified above were supported by phylogenetic analyses (Figure 3).

Group I could be further subdivided into three orthology groups, named group Ia, group Ib, and group Ic, respectively. Among these groups, group Ia contained genes from different plants ranging from green algae (e.g., C. braunii) to higher plants (e.g., A. thaliana and S. lycopersicum), group Ib was composed of genes from plants ranging from mosses to higher plants, and group Ic only consisted of homologs from dicots (Figure 3). Subcellular localization predictions suggested that all the members from group Ia and group Ib reside in the chloroplast and cytosol, respectively, while homologs from group Ic were predicted to be localized in either the cytosol or the peroxisome (Figure 3). Although the relationship between group Ia, group Ib, and group Ic could not be determined due to the lack of strong bootstrap support, our results indicate an early origin for genes from group I in the most recent common ancestor (MRCA) of plants and that at least two duplication events occurred during the plants’ evolution.

Furthermore, according to our phylogenetic analyses, group II was a monophyletic group. Subcellular localization predictions suggested that most of its members reside in the chloroplast. A detailed examination found that group II contained genes from several major lineages of green plants, including algae, mosses, and seed plants (Figure 3), suggesting that they originated in the ancestors of green plants.

In addition, Figure 3 displays phylogenetic relationships of Cu/Zn SOD proteins from group Ib where there are examples of lineage-specific gene expansions, as was the case for the ferns (A. filiculoides, S. cucullate, and S. moellendorffii) and monocot (O. sativa, S. bicolor, Z. mays, and B. distachyon) (Figure 3). In addition, species-specific expansions were also observed in group Ia (A. comosus) and group Ib (E. grandis, M. esculenta, D. carota, and A. comosus) (Figure 3). Usually, these can result in the expansion of one functional class of Cu/Zn SOD proteins.

In this study, we were able to comprehensively investigate the evolutionary landscape of Cu/Zn SOD proteins by comparing genome-wide samplings of Cu/Zn SOD genes from different plant species ranging from lower to higher plants. Phylogenetic analyses showed that the Cu/Zn SODs from the tested plant species were apparently separated into two groups (group I and group II) with strong bootstrap support (Figure 3), indicating an independent evolution of the Cu/Zn SOD genes in plant species. Notably, all Cu/Zn SOD genes without a heavy metal-associated domain formed a clade (group I), which was further subdivided into three subgroups (Ia, Ib, and Ic), while all Cu/Zn SOD genes with a heavy metal-associated domain formed a single gene clade (group II) with strong bootstrap support and were monophyletic in different plant species (Figure 3). This suggests that the two groups of Cu/Zn SOD genes may have originated from different common ancestors and evolved independently. We thus demonstrated the independent evolution of Cu/Zn SOD genes in different plant species that may have had different origins.

Neofunctionalization (co-option of new paralogues into novel functions) is a common outcome of gene duplication events [39] and may underlie adaptations to specific ecological niches [40,41,42,43]. Additionally, it is possible that subfunctionalization is a key driving force for the retention of multiple family members [44]. At present, a multitude of functions have been ascribed to Cu/Zn SOD proteins; therefore, gene duplication and retention may represent the partitioning of these functions between numbers of genes.

### 2.4. Structural Feature of Cu/Zn SOD Proteins: Loss of Intron and Signal Peptide

**Figure 4:**
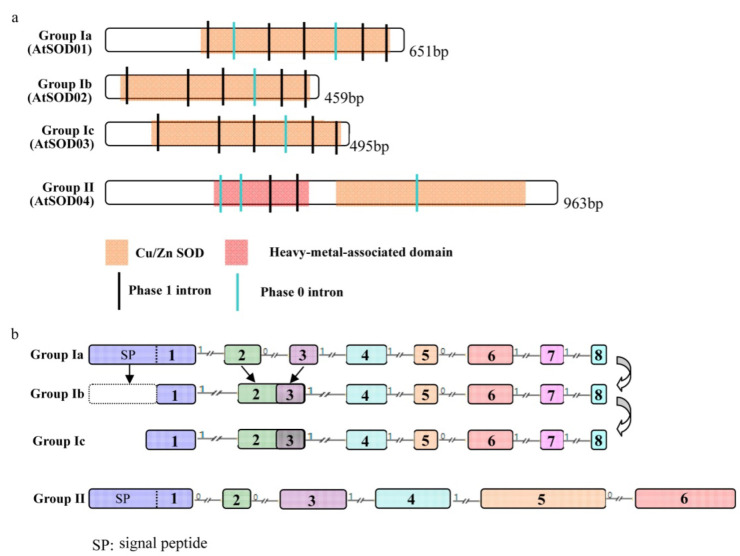
Structure and sequence features of conserved Cu/Zn SOD genes. (**a**) Gene structure and protein motif. The structure of an Arabidopsis thaliana gene (indicated on the left) is shown as an example for each subgroup (in parenthesis on the left). Protein motifs are shown as colored boxes, whereas introns of different phases are shown as colored vertical lines. (**b**) Detailed analyses of gene structure of Cu/Zn SOD genes. The numbers indicate different exons. The introns are drawn to scale. The arrows represent stepwise duplication of Cu/Zn SOD genes from group I during plant evolution.

To analyze the mechanisms of the structural features of Cu/Zn SOD genes, the exon/intron structures of Cu/Zn SOD genes were compared across all the tested plant lineages. The exon/intron structures were investigated using the online tool “Gene Structure Display Server” with both encoding sequences (CDS) and genomic sequences [38]. Figure 4a shows a detailed illustration of the relative lengths of introns and conservation of the corresponding exon sequences within each of the subgroups in the plants. Notably, although the members of the Cu/Zn SOD gene family exhibited differences in intron number and intron length, the intron positions and intron phases were conserved (Figure 4a). As for the numbers of introns, the Cu/Zn SOD genes in group Ia contained seven introns while those in groups Ib and Ic contained six introns (Figure 4a). All the introns presented protein motifs for the Cu/Zn SOD genes. Two intron phases (phase 1 and phase 0) were present in all the Cu/Zn SOD genes, while intron phase 2 was not found (Figure 4a). Interestingly, the Cu/Zn SOD genes in the oldest subfamily, group Ia, contained the greatest numbers of introns, while those in the youngest subfamily, groups Ib and Ic, contained the fewest introns (Figure 4a). These findings, together with the phylogenetic trees, indicate that an intron loss event occurred during the structural evolution of the Cu/Zn SOD gene family from green algae to angiosperms. In group II, a total of five different introns were found within all the genes from the group across the different lineage species (Figure 4a). The results show that, unlike the evolutionary patterns of group I, the exon/intron structures of Cu/Zn SOD genes from group II were high conserved.

We also found that the length of the first exon varied between groups Ia, Ib, and Ic (Figure 4b). Further analysis showed that the 5′-terminal of the first exon in Cu/Zn SOD genes from groups Ib and Ic was reduced compared to those from group Ia, leading to a loss of signal peptides and indicating that shifts in subcellular localization had occurred during the process of plant evolution.

### 2.5. Protein–Protein Interaction Networks of Potential Cn/Zn SODs

**Figure 5:**
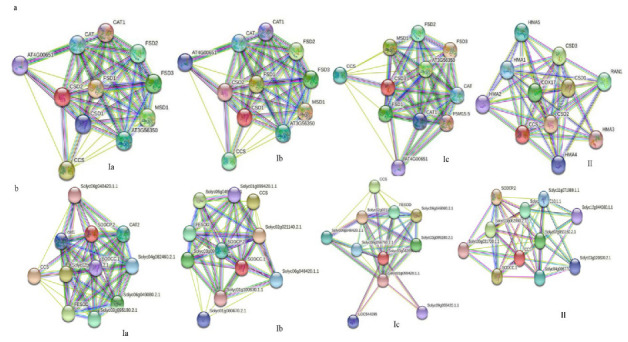
Potential protein–protein interaction network of plant Cu/ZnSODs. (**a**) Arabidopsis thaliana. (**b**) Solanum lycopersicum. The higher the interaction coefficient, the thicker the line between proteins is, and vice versa.

Next, we further analyzed the potential protein–protein interactions of Cu/Zn SODs from A. thaliana and tomato using the online web tool (https://string-db.org/, accessed on 14 December 2021). The results showed that three Cu/Zn SOD proteins (AtCSD1, AtCSD2, and AtCSD3) from A. thaliana are associated with known SOD proteins (Fe-SOD, Mn-SOD) in the interaction network. Moreover, a strong interaction between CSDs and the copper chaperone for superoxide dismutase (CCS) was observed. It is well-known that CCS is responsible for the transfer of Cu^2+^ into the cytoplasm, which increases the concentration of Cu^2+^ in the cytoplasm, thereby promoting the expression of CSD1 [45]. Additionally, we also found a high level of interaction between CSDs and other antioxidant enzymes, such as catalase (CAT). The findings imply that, in plant antioxidant enzyme systems, these two types of enzymes might respond to various biotic and abiotic stresses in a synergetic manner (Figure 5a). Interestingly, a similar phenomenon also occurred in tomato (Figure 5b).

### 2.6. Expression of Cu/Zn SOD Genes during Plant Development

**Figure 6:**
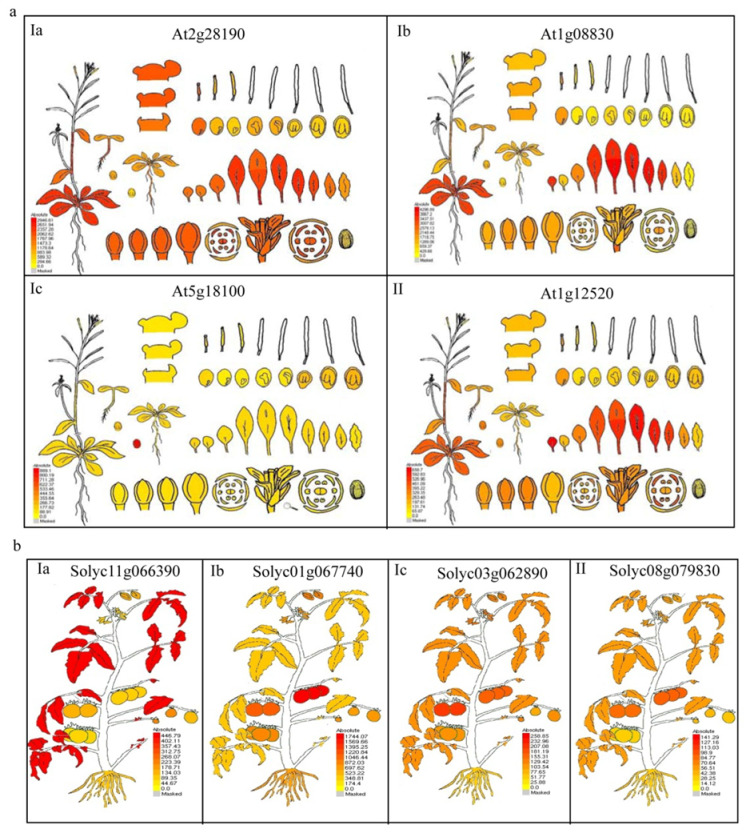
Expressions of Cu/Zn SOD genes during the growth and development of *Arabidopsis thaliana* (**a**) and *Solanum lycopersicum* (**b**). The gene is indicated in each graph. Detailed expression levels in various tissues and organs are indicated in a database online (http://bar.utoronto.ca/efp/cgi-bin/efpWeb.cgi, accessed on 14 December 2021).

Next, differences in gene expression in plant organs were analyzed using online tools (http://bar.utoronto.ca/, accessed on 14 December 2021). Under normal conditions, all the Cu/Zn SOD genes from A. thaliana and S. lycopersicum were constitutively expressed in different tissues (Figure 6a,b). In A. thaliana, three Cu/Zn SOD genes from groups Ia, Ib, and II, respectively, were found to be expressed in all organs tested and were preferentially expressed in leaves; however, the Cu/Zn SOD gene from group Ic had tissue-specific or preferential expression patterns in seeds (fruit). In tomato, similar expression patterns of Cu/Zn SOD genes from different groups were observed. For example, the preferential expression of the Cu/Zn SOD genes from group I and group II was observed for leaves, but the Cu/Zn SOD genes from group Ic were preferentially expressed in fruit. We found that the Cu/Zn SOD genes from the same subgroups had similar expression patterns, which suggests potential functional conservation during the long evolution process.

### 2.7. Expression of Plant Cu/Zn SOD Genes Suggests Possible Roles in Abiotic Stresses

**Figure 7:**
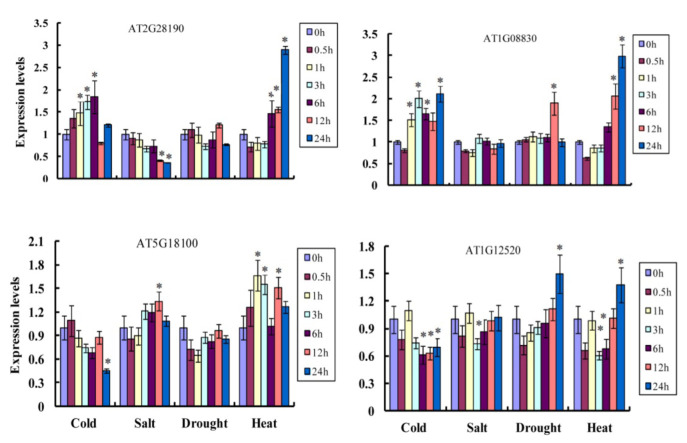
The expression pattern of Cu/Zn SOD genes in Arabidopsis thaliana under different types of stresses, including cold, heat, salt, and drought. Statistically significant differences are indicated *p* < 0.05 by star (*) (Student’s *t*-test).

Based on RNA-seq data for Arabidopsis thaliana, we analyzed the stress responsiveness and expression patterns of Cu/Zn SOD genes under four types of abiotic stress (cold, heat, salt, and drought). The two Cu/Zn SOD genes (groups Ia and Ib) were up-regulated under low temperature, and the remaining two genes (groups Ic and II) were down-regulated. For the salt treatment, the Cu/Zn SOD genes from groups Ia and Ic showed down- and up-regulation, respectively, while Cu/Zn SOD genes from groups Ib and II were not induced. For the drought treatment, expressions of the Cu/Zn SOD genes from groups Ia and Ic were almost consistent, while the remaining two Cu/Zn SOD genes from groups Ib and II were markedly induced. Interestingly, all the Cu/Zn SOD genes showed up-regulation under the heat treatment condition. These results suggest that plant Cu/Zn SOD genes have a potential role in abiotic stresses (Figure 7).

## 3. Discussion

### 3.1. High Conservation of Cu/Zn SOD Gene Family in Plant Species

It was well known that, as the first line of defense in the antioxidant system, SODs play vital roles in protecting plant cells from oxidative damage by catalyzing the dismutation of superoxide free radicals [46]. Thus, the identification and analysis of all the members of the SOD gene family is important to provide a basis for the enhancement of stress resistance in plants. The availability of multiple plant genomes has made it possible to identify all SOD genes at the whole-genome level. Previously, the plant SOD gene family was identified in several model plant species, such as banana [47], wheat [48], *A. thaliana* [32], and tomato [33], but little was reported from the range of lower to higher plant species. Here, using the bioinformatic method, a genome-wide survey of the Cu/Zn SOD gene family in different plant species ranging from lower to higher plants at the whole-genome level was performed. The results will enrich our understanding of the plant SOD gene family and provide a foundation for understanding their evolutionary patterns. Additionally, only a few members of the Cu/Zn SOD gene family were identified in barley, banana, *A. thaliana*, and tomato [32,33,49]. In our study, roughly two to seven members were found in different plant species. These results suggest that the Cu/Zn SOD genes could be encoded by a relatively small gene family with several members, from lower plants (e.g., *C. braunii*) to higher plants (e.g., *A. thaliana* and *S. lycopersicum*). However, multiple members (17) of the Cu/Zn gene family, also reported in wheat with whole-genome duplication, indicate that whole-genome duplication, a common phenomenon in plant genomes, drove the amplification of the Cu/Zn SOD gene family, and this could have led to an increase in the number of genes, which is of key significance for plant evolution.

### 3.2. Contrasting Evolutionary Histories between Group I and Group II

In this study, we found that Cu/Zn SOD genes from these two groups had strikingly different evolutionary patterns. Group I genes expanded during the histories of the different plants tested (Figure 3). Following gene duplication, members of three different subgroups (group Ia, group Ib, and group Ic) likely experienced functional divergence, as supported by expression analyses. Unlike group I genes, Cu/Zn SOD genes from group II are single- or low-copy genes. The stably maintained single- or low-copy numbers for these genes suggest functional conservation during plant evolution. Furthermore, we found that the members from group II had similar gene structures and sequence features (Figure 4). Therefore, our findings further support the idea that these genes with ancient origins still retain rather conserved functions.

### 3.3. Loss of Introns and Signal Peptides: The Possible Functional Divergence

Generally, gene structures contain different numbers of exons and introns [50], and the number of introns is closely related to the complexity of the eukaryote’s genome, with most eukaryotes having two or more introns [51]. In this study, we found that the intron numbers of Cu/Zn SOD genes were quite different, varying between five and seven for group I and group II. This finding was in accordance with the previous view that there are no similar SOD gene structures in different species [46]. Previous researchers reported that exon/intron gain/loss can be attributed to structural divergences, which bring about orthologous/paralogous genes evolving different numbers of introns [52]. In our research, based on the phylogenetic relationship, we inferred that at least two lineage-specific expansions had occurred in group I during the course of evolution, and group Ib and group Ic were produced by group Ia duplication. Among the three orthology subgroups, an intron loss event was found between group Ia and group Ib. However, the gene structures were the same between groups Ib and Ic. In addition, as an N-terminal signal peptide indicative of localization to the chloroplast was present in group Ia, while group Ib lacked this domain and appeared to be localized to the cytosol, an event involving the loss of a signal peptide seems to have occurred in the course of evolution. However, group Ic possessed a relatively long N-terminal sequence, which is indicative of localization at the cytosol or peroxisome; therefore, as well as the event involving the loss of the signal peptide, the sequence-variation-encoded signal peptide could have appeared over the long course of evolution, transferring the subcellular localization from the chloroplast to the peroxisome during plant evolution. All these results suggest the possible functional divergence of all the Cu/Zn SOD genes.

### 3.4. Proposed Evolutionary History of the Cu/Zn Gene Family in Plants

**Figure 8:**
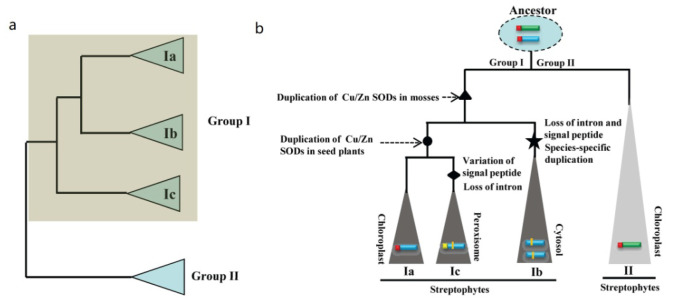
Evolutionary history and duplication timing of the Cu/Zn SOD gene family in plant species as proposed. (**a**) Tree summarizing our current understanding of the phylogenetic relationships based on our analyses. Two groups (group I and group II) were identified. (**b**) Evolutionary scenario explaining how a single-tandem duplication and subsequent large-scale duplications expanded the Ia, Ib, Ic, and II lineages.

Our phylogenetic study revealed that two Cu/Zn SOD members of *C. braunii* (Charophyte) are found in groups I and II, indicating that Cu/Zn SOD genes from these groups originated independently (Figure 3 and Figure 8a). As a result, the minimum number of Cu/Zn SOD genes in the last common ancestor of groups I and II is one (Figure 8b). Only one member of the Cu/Zn SOD gene family from group II was detected in each of the plant species studied, indicating that the genes have been better conserved over time. In group I, however, numerous members of the Cu/Zn SOD gene family were discovered. Further investigation revealed that the four members of the *P. patens* Cu/Zn gene family constitute two pairs of highly identical genes in subgroups Ia and Ib, implying that a previous two-member gene family was duplicated. As a result, we deduced that the Cu/Zn SOD genes from group I experienced their first significant expansion (in terms of both number and diversity) after land colonization. Losses of the second intron and signal peptide for group Ib were found after the gene growth in group I, indicating a shift in subcellular localization from chloroplast to cytosol. There was also evidence of species-specific duplication (Figure 8b). The second expansion of group I occurred in seed plants, resulting in group Ic, which had a single member with Cu/Zn SODs from the plant species studied. The loss of the second intron in Cu/Zn SOD genes for group Ic was also discovered; however, changes in the signal peptide sequence were observed, implying subcellular shifts from chloroplast to peroxisome (Figure 8b).

### 3.5. The Cu/Zn SOD Genes Play Vital Roles in Response to Abiotic Stress

Accumulation of ROS caused by abiotic stresses, such as cold and drought, could pose threats to plant yield and quality. SODs, the first line of defense against oxidative damage, are involved in ROS scavenging in response to various abiotic stresses in different plants [53]. In this study, the expression levels of most SOD genes revealed dynamic trends at certain times from 0 h to 24 h under abiotic stresses (cold, heat, salt, and drought). Evidence for this explanation in *S. lycopersicum* [33], *G. hirusutm* [36], *Brassica juncea,* and *B. rapa* [54] is accumulating. Additionally, it has been reported that enhanced expression of squash SOD genes is involved in ROS detoxification in response to these stresses [55]. In the present study, we observed different expression patterns for Cu/Zn SOD genes in response to these abiotic stresses, indicating potential functional divergence.

## 4. Materials and Methods

### 4.1. Retrieval Databases for Cu/Zn SOD Gene Family in Representative Plant Species

In this paper, different plant databases were used to identify potential Cu/Zn SOD genes. These databases are provided in Table 1.

### 4.2. Identification of Plant Cu/Zn SOD Gene Family Using Bioinformatic Methods

A systematic search for Cu/Zn SOD proteins was conducted using the BLASTP algorithm with a threshold e-value of 1 × 10^5^. Cu/Zn SOD protein sequences from *A. thaliana* (AT1G08830.1/AtCSD1, AT2G28190.1/AtCSD2, AT5G18100.1/AtCSD3, AT1G12520/AtCSS) were used as queries. Further, the local HMMER 3.1 web server (available online: http://www.hmmer.org/, accessed on 14 December 2021) was used to search for the Cu/Zn SOD genes with default parameters. Then, the HMM file of the Sod_Cu (PF00080.21) with Cu/Zn SOD genes was downloaded from the Pfam protein domain database.

### 4.3. Analysis of Gene Structure and Potential Protein Interaction of Cu/Zn SOD Proteins

Intron/exon configurations of Cu/Zn SOD genes were determined with Gene Structure Display Server 6 for both coding sequences and genomic sequences [56]. Gene structure analysis and intron positions were retrieved from genomic GFF files and converted into the relative coordinates of the open reading frame (ORF). Phase 0 is for an intron between two codons, phase 1 is between the first and second nucleotides of a codon, and phase 2 is for other cases. The interactions among Cu/Zn SOD proteins were determined using STRING (http://string-db.org/, accessed on 14 December 2021) with a confidence score >0.9.

### 4.4. Phylogenetic Tree Construction for Cu/Zn SODs

To investigate the phylogenetic relationships of Cu/Zn SOD genes, all Cu/Zn SOD protein sequences from 23 representative plant species were used to construct phylogenetic trees ranging from lower to higher plant species. Multiple sequence alignments of these Cu/Zn SOD amino acid sequences were performed with ClustalX [57] using default parameters. A phylogenetic tree was constructed using the software MEGA5.1 with the neighbor-joining method [58]. In the phylogenetic tree, the degree of support for a particular grouping pattern was evaluated using a bootstrap value (1000 replicates), and other parameters were all set to default [59]. The tree was viewed using the iTOL web tool (https://itol.embl.de/, accessed on 14 December 2021).

### 4.5. Prediction of Subcellular Localization and Conserved Motifs of Cu/Zn SOD Proteins

The subcellular localization of Cu/Zn SOD proteins was predicted using the WoLF PSORT server (https://wolfpsort.hgc.jp/, accessed on 14 December 2021). The conserved motifs for the Cu/Zn SOD protein sequences were identified using the MEME website (https://meme-suite.org/meme/db/motifs, accessed on 14 December 2021).

### 4.6. Expression Patterns of Cu/Zn SOD Genes Based on Affymetrix Microarray Data

For tissue-specific expression analysis, transcription data for the genome-wide gene expression of *A. thaliana* and tomato were downloaded from http://bar.utoronto.ca/, accessed on 14 December 2021. A total of 47 different tissues or development periods were selected (http://bar.utoronto.ca/efp/cgi-bin/efpWeb.cgi, accessed on 14 December 2021). For abiotic stress treatments, seeds of wild-type *A. thaliana* (col-0) were sown on rafts in Magenta boxes containing MS-Agar-media. After 2 days in the cold room, the boxes were transferred to the long-day chamber. Long-day conditions were 16/8 h light/dark and 50% humidity. At day 16, stress treatments started with a 3 h light period; samples were taken at 0.5, 1, 3, 6, 12, and 24 h after treatment. Four types of abiotic stresses were included (cold—continuous 4 °C on crushed ice in a cold chamber, 150 Mm NaCl salt; drought—rafts were exposed to the air stream for 15 min with loss of approximately 10% fresh weight; heat—3 h at 38 °C followed by recovery at 25 °C). Biologically different samples were studied in triplicate. Three replications were examined to determine expression levels. A paired t-test was calculated with significance set at *p* ≤ 0.05.

## 5. Conclusions

In this study, we found that the Cu/Zn SOD gene family in lower to higher plant species could be divided into two well-conserved subgroups (group I and group II), which originated from different ancestors of green plants. In contrast to group II, lineage-specific expansion contributed to the size of the group I gene family, and three subgroups (group Ia, group Ib, and group Ic) formed in the plants. The gene structure analysis indicated that successive single-intron loss, signal peptide loss, and sequence variation in the signal peptide played vital roles in the evolution of Cu/Zn SOD genes. Additionally, the expression analysis for the Cu/Zn SOD genes suggested a functional divergence of these genes in response to abiotic stresses (cold, heat, salt, and drought). These results may provide valuable information for future studies on the evolution and function of this gene family.

## Figures and Tables

**Table 1:** The databases for the different plant species used in this study.

	Plant Species	Databases
1	*Amborella trichopoda*	https://phytozome-next.jgi.doe.gov/info/Atrichopoda_v1_0, accessed on 14 December 2021
2	*Ananas comosus*	https://phytozome-next.jgi.doe.gov/info/Acomosus_v3, accessed on 14 December 2021
3	*Aquilegia coerulea*	https://phytozome-next.jgi.doe.gov/info/Acoerulea_v3_1, accessed on 14 December 2021
4	*Azolla filiculoides*	https://www.fernbase.org/, accessed on 14 December 2021
5	*Brachypodium distachyon*	https://phytozome-next.jgi.doe.gov/info/Bdistachyon_v3_2, accessed on 14 December 2021
6	*Capsicum annuum*	http://www.hnivr.org/, accessed on 14 December 2021
7	*Chara braunii*	https://bioinformatics.psb.ugent.be/orcae/overview/Chbra, accessed on 14 December 2021
8	*Cucumis sativus*	https://phytozome-next.jgi.doe.gov/info/Csativus_v1_0, accessed on 14 December 2021
9	*Daucus carota*	https://phytozome-next.jgi.doe.gov/info/Dcarota_v2_0, accessed on 14 December 2021
10	*Eucalyptus grandis*	https://phytozome-next.jgi.doe.gov/info/Egrandis_v2_0, accessed on 14 December 2021
11	*Marchantia polymorpha*	https://phytozome-next.jgi.doe.gov/info/Mpolymorpha_v3_1, accessed on 14 December 2021
12	*Manihot esculenta*	https://phytozome-next.jgi.doe.gov/info/Mesculenta_v6_1, accessed on 14 December 2021
13	*Medicago truncatula*	https://phytozome-next.jgi.doe.gov/info/Mtruncatula_Mt4_0v1, accessed on 14 December 2021
14	*Oryza sativa*	https://phytozome-next.jgi.doe.gov/info/Osativa_v7_0, accessed on 14 December 2021
15	*Phaseolus vulgaris*	https://phytozome-next.jgi.doe.gov/info/PvulgarisUI111_v1_1, accessed on 14 December 2021
16	*Physcomitrium patens*	https://phytozome-next.jgi.doe.gov/info/Ppatens_v3_3, accessed on 14 December 2021
17	*Ricinus communis*	https://phytozome-next.jgi.doe.gov/info/Rcommunis_v0_1, accessed on 14 December 2021
18	*Salvinia cucullata*	https://www.fernbase.org/, accessed on 14 December 2021
19	*Selaginella moellendorffii*	https://phytozome-next.jgi.doe.gov/info/Smoellendorffii_v1_0, accessed on 14 December 2021
20	*Solanum lycopersicum*	https://phytozome-next.jgi.doe.gov/info/Slycopersicum_ITAG4_0, accessed on 14 December 2021
21	*Sorghum bicolor*	https://phytozome-next.jgi.doe.gov/info/SbicolorRTx430_v2_1, accessed on 14 December 2021
22	*Volvox carteri*	https://phytozome-next.jgi.doe.gov/info/Vcarteri_v2_1, accessed on 14 December 2021
23	*Zea mays*	https://phytozome-next.jgi.doe.gov/info/ZmaysPH207_v1_1, accessed on 14 December 2021

## Data Availability

The data used for the analysis in this study are available within the article and Supplementary Materials.

## Supplementary Materials

The following are available online at https://www.mdpi.com/article/10.3390/ijms23137082/s1.

## References

[1] Apel K., Hirt H. (2004). Reactive oxygen species: Metabolism, oxidative stress, and signal transduction. Annu. Rev. Plant Biol..

[2] Foyer C.H., Noctor G. (2005). Redox homeostasis and antioxidant signaling: A metabolic interface between stress perception and physiological responses. Plant Cell.

[3] Gill S.S., Tuteja N. (2010). Reactive oxygen species and antioxidant machinery in abiotic stress tolerance in crop plants. Plant Physiol. Biochem..

[4] Gill S.S., Khan N.A., Anjum N.A., Tuteja N., Anjum N.A., Lopez-Lauri F. (2011). Amelioration of cadmium stress in crop plants by nutrients management: Morphological, physiological and biochemical aspects. Plant Nutrition and Abiotic Stress Tolerance III.

[5] Miller G., Suzuki N., Ciftci-Yilmaz S., Mittler R. (2010). Reactive oxygen species homeostasis and signalling during drought and salinity stresses. Plant Cell Environ..

[6] Scandalios J.G. (1993). Oxygen stress and superoxide dismutases. Plant Physiol..

[7] Huseynova I.M. (2012). Photosynthetic characteristics and enzymatic antioxidant capacity of leaves from wheat cultivars exposed to drought. Biochim. Biophys. Acta.

[8] Kehrer J.P. (2000). The Haber-Weiss reaction and mechanisms of toxicity. Toxicology.

[9] Racchi M.L., Bagnoli F., Balla I., Danti S. (2001). Differential activity of catalase and superoxide dismutase in seedlings and in vitro micropropagated oak (*Quercus robur* L.). Plant Cell Rep..

[10] Puget K., Michelson A.M. (1974). Isolation of a new copper-containing superoxide dismutase bacteriocuprein. Biochem. Biophys. Res. Commun..

[11] Deshazer D., Barnnan J.D., Moran M.J., Friedman R.L. (1994). Characterization of the gene encoding superoxide dismutase of *Bordetella pertussis* and construction of a SOD-deficient mutant. Gene.

[12] del Río L.A., Sandalio L.M., Corpas F.J., López-Huertas E., Palma J.M., Pastori G.M. (1998). Activated oxygen-mediated metabolic functions of leaf peroxisomes. Plant Physiol..

[13] Mishra S., Jha A.B., Dubey R.S. (2011). Arsenite treatment induces oxidative stress, upregulates antioxidant system, and causes phytochelatin synthesis in rice seedlings. Protoplasma.

[14] Zlatev Z.S., Lidon F.C., Ramalho J.C., Yordanov I.T. (2006). Comparison of resistance to drought of three bean cultivars. Biol. Plant..

[15] Sharma P., Dubey R.S. (2005). Drought induces oxidative stress and enhances the activities of antioxidant enzymes in growing rice seedlings. Plant Growth Regul..

[16] Sharma P., Dubey R.S. (2005). Modulation of nitrate reductase activity in rice seedlings under aluminium toxicity and water stress: Role of *osmolytes* as enzyme protectant. J. Plant Physiol..

[17] Wang C.Q., Li R.C. (2008). Enhancement of superoxide dismutase activity in the leaves of white clover (*Trifolium repens* L.) in response to polyethylene glycol-induced water stress. Acta Physiol. Plant..

[18] Kukreja S., Nandwal A.S., Kumar N., Sharma S.K., Unvi V., Sharma P.K. (2005). Plant water status, H_2_O_2_ scavenging enzymes, ethylene evolution and membrane integrity of *Cicer arietinum* roots as affected by salinity. Biol. Plant..

[19] Gapiñska M., Skłodowska M., Gabara B. (2008). Effect of short-and long-term salinity on the activities of antioxidative enzymes and lipid peroxidation in tomato roots. Acta Physiol. Plant..

[20] Sharma P., Jha A.B., Dubey R.S., Pessarakli M. (2012). Reactive oxygen species, oxidative damage, and antioxidative defense mechanism in plants under stressful conditions. J. Bot..

[21] Zaefyzadeh M., Quliyev R.A., Babayeva S.M., Abbasov M.A. (2009). The effect of the interaction between genotypes and drought stress on the superoxide dismutase and chlorophyll content in durum wheat landraces. Turk. J. Biol..

[22] Perl-Treves R., Nacmias B., Aviv D., Zeelon E.P., Galun E. (1988). Isolation of two cDNA clones from tomato containing two different superoxide dismutase sequences. Plant Mol. Biol..

[23] Perl-Treves R., Abu-Abied M., Magal N., Galun E., Zamir D. (1990). Genetic mapping of tomato cDNA clones encoding the chloroplastic and the cytosolic isozymes of superoxide dismutase. Biochem. Genet..

[24] Perl-Treves R., Galun E. (1991). The tomato Cu,Zn superoxide dismutase genes are developmentally regulated and respond to light and stress. Plant Mol. Biol..

[25] Wu J., Zhang J., Li X., Xu J.J., Wang L. (2016). Identification and characterization of a Put Cu/Zn SOD gene from *Puccinellia tenuiflora* (Turcz.) Scribn. et Merr. Plant Growth Regul..

[26] Negi N.P., Shrivastava D.C., Sharma V., Sarin N.B. (2015). Overexpression of Cu/Zn SOD from *Arachis hypogaea* alleviates salinity and drought stress in tobacco. Plant Cell Rep..

[27] Jing X., Hou P., Lu Y., Deng S., Li N., Zhao R., Sun J., Wang Y., Han Y., Lang T. (2015). Overexpression of copper/zinc superoxide dismutase from mangrove *Kandelia candel* in tobacco enhances salinity tolerance by the reduction of reactive oxygen species in chloroplast. Front. Plant Sci..

[28] Wang M., Zhao X., Xiao Z., Yin X., Xing T., Xia G. (2016). A wheat superoxide dismutase gene TaSOD2 enhances salt resistance through modulating redox homeostasis by promoting NADPH oxidase activity. Plant Mol. Biol..

[29] Li Z., Han X., Song X., Zhang Y., Jiang J., Han Q., Liu M., Qiao G., Zhuo R. (2017). Overexpressing the *Sedum alfredii* Cu/Zn superoxide dismutase increased resistance to oxidative stress in transgenic *Arabidopsis*. Front. Plant Sci..

[30] Perl A., Perl-Treves R., Galili S., Aviv D., Shalgi E., Malkin S., Galun E. (1993). Enhanced oxidative-stress defense in transgenic potato expressing tomato Cu, Zn superoxide dismutases. Theor. Appl. Genet..

[31] Bernal A., Torres J., Reyes A., Rosado A. (2009). Exogenous auxin regulates H_2_O_2_ metabolism in roots of tomato (*Lycopersicon esculentum* mill.) seedlings affecting the expression and activity of CuZn-superoxide dismutase, catalase, and peroxidase. Acta Physiol. Plant..

[32] Kliebenstein D.J., Monde R.A., Last R.L. (1998). Superoxide dismutase in Arabidopsis: An eclectic enzyme family with disparate regulation and protein localization. Plant Physiol..

[33] Feng K., Yu J., Cheng Y., Ruan M., Wang R., Ye Q., Zhou G., Li Z., Yao Z., Yang Y. (2016). The SOD gene family in tomato: Identification, phylogenetic relationships, and expression patterns. Front. Plant Sci..

[34] Zhou Y., Hu L., Wu H., Jiang L., Liu S. (2017). Genome-wide identification and transcriptional expression analysis of cucumber superoxide dismutase (SOD) family in response to various abiotic stresses. Int. J. Genom..

[35] Wang W., Xia M., Chen J., Deng F., Yuan R., Zhang X., Shen F. (2016). Genome-wide analysis of superoxide dismutase gene family in *Gossypium raimondii* and *G. arboreum*. Plant Gene.

[36] Wang W., Zhang X., Deng F., Yuan R., Shen F. (2017). Genome-wide characterization and expression analyses of superoxide dismutase (SOD) genes in *Gossypium hirsutum*. BMC Genom..

[37] Zhang J., Li B., Yang Y., Hu W., Chen F., Xie L., Fan L. (2016). Genome-wide characterization and expression profiles of the superoxide dismutase gene family in *Gossypium*. Int. J. Genom..

[38] Li J., Lee J.Y., Liao L. (2021). A new algorithm to train hidden Markov models for biological sequences with partial labels. BMC Bioinform..

[39] Prud’homme B., Lartillot N., Balavoine G., Adoutte A., Veroort M. (2002). Phylogenetic analysis of the *Wnt* gene family: Insights from *lophotrochozoan* members. Curr. Biol..

[40] Conant G.C., Wolfe K.H. (2008). Turning a hobby into a job: How duplicated genes find new functions. Nat. Rev. Genet..

[41] Chung H.R., Lohr U., Jackle H. (2007). Lineage-specific expansion of the zinc finger associated domain ZAD. Mol. Biol. Evol..

[42] Lespinet O., Wolf Y.I., Koonin E.V., Aravind L. (2002). The role of lineage-specific gene family expansion in the evolution of eukaryotes. Genome Res..

[43] Wade N.M., Tollenaere A., Hall M.R., Degnan B.M. (2009). Evolution of a novel carotenoid-binding protein responsible for crustacean shell color. Mol. Biol. Evol..

[44] Aguilera F., Mcdougall C., Degnan B.M. (2013). Origin, evolution and classification of type-3 copper proteins: Lineage-specific gene expansions and losses across the Metazoa. BMC Evol. Biol..

[45] Casareno R.L., Waggoner D., Gitlin J.D. (1998). The copper chaperone CCS directly interacts with copper/zinc superoxide dismutase. J. Biol. Chem..

[46] Fink R.C., Scandalios J.G. (2002). Molecular evolution and structure-function relationships of the superoxide dismutase gene families in angiosperms and their relationship to other eukaryotic and prokaryotic superoxide dismutases. Arch. Biochem. Biophys..

[47] Feng X., Lai Z., Lin Y., Lai G., Lian C. (2015). Genome-wide identification and characterization of the superoxide dismutase gene family in *Musa acuminata* cv. Tianbaojiao (AAA group). BMC Genom..

[48] Jiang W.Q., Yang L., He Y.Q., Zhang H.T., Li W., Chen H.G., Ma D.F., Yin J.L. (2019). Genome-wide identification and transcriptional expression analysis of superoxide dismutase (SOD) family in wheat (*Triticum aestivum*). PeerJ.

[49] Zhang X., Zhang L.T., Chen Y., Wang S.Y., Fang Y.X., Zhang X.Q., Wu Y.H., Xue D.W. (2021). Genome-wide identification of the SOD gene family and expression analysis under drought and salt stress in barley. Plant Growth Regul..

[50] Sakharkar M.K., Chow V.T.K., Kangueane P. (2004). Distributions of exons and introns in the human genome. Silico Biol..

[51] Rogozin I.B., Sverdlov A.V., Babenko V.N., Koonin E.V. (2005). Analysis of evolution of exon-intron structure of eukaryotic genes. Brief. Bioinform..

[52] Xu G., Guo C., Shan H., Kong H. (2012). Divergence of duplicate genes in exon-intron structure. Proc. Natl. Acad. Sci. USA.

[53] Gill S.S., Anjum N.A., Gill R., Yadav S., Hasanuzzaman M., Fujita M., Mishra P., Sabat S., Tuteja N. (2015). Superoxide dismutase-mentor of abiotic stress tolerance in crop plants. Environ. Sci. Pollut. Res..

[54] Verma D., Lakhanpal N., Singh K. (2019). Genome-wide identification and characterization of abiotic-stress responsive SOD (superoxide dismutase) gene family in *Brassica juncea* and *B. rapa*. BMC Genom..

[55] Chiang C.M., Kuo W.S., Lin K.H. (2014). Cloning and gene expression analysis of sponge gourd ascorbate peroxidase gene and winter squash superoxide dismutase gene under respective flooding and chilling stresses. Hortic. Environ. Biotechnol..

[56] Hu B., Jin J., Guo A.Y., Zhang H., Luo J., Gao G. (2014). GSDS 2.0: An upgraded gene feature visualization server. Bioinformatics.

[57] Higgins D.G., Thompson J.D., Gibson T.J. (1996). Using CLUSTAL for multiple sequence alignments. Methods Enzymol..

[58] Tamura K., Peterson D., Peterson N., Stecher G., Nei M., Kumar S. (2011). MEGA5: Molecular, evolutionary, genetics, analysis, using maximum, likelihood, evolutionary, distance, and maximum, parsimony, methods. Mol. Biol. Evol..

[59] Filiz E., Tombuloğlu H. (2014). Genome-wide distribution of superoxide dismutase (SOD) gene families in Sorghum bicolor. Turk. J. Biol..

